# Glycemic and Metabolic Effects of Two Long Bouts of Moderate-Intensity Exercise in Men with Normal Glucose Tolerance or Type 2 Diabetes

**DOI:** 10.3389/fendo.2017.00154

**Published:** 2017-07-11

**Authors:** Saeed Reza Eshghi, Kevin Fletcher, Étienne Myette-Côté, Cody Durrer, Raniah Q. Gabr, Jonathan P. Little, Peter Senior, Craig Steinback, Margie H. Davenport, Gordon J. Bell, Dion R. Brocks, Normand G. Boulé

**Affiliations:** ^1^Faculty of Physical Education and Recreation, University of Alberta, Edmonton, AB, Canada; ^2^Alberta Diabetes Institute, University of Alberta, Edmonton, AB, Canada; ^3^School of Health and Exercise Sciences, University of British Columbia, Kelowna, BC, Canada; ^4^National Organization for Drug Control and Research (NODCAR), Giza, Egypt; ^5^Division of Endocrinology and Metabolism, Department of Medicine, Faculty of Medicine, University of Alberta, Edmonton, AB, Canada; ^6^Faculty of Pharmacy and Pharmaceutical Sciences, University of Alberta, Edmonton, AB, Canada

**Keywords:** aerobic exercise, glucose tolerance, glucagon, insulin, glucagon-like peptide-1, glucose-dependent insulinotropic peptide

## Abstract

**Background:**

The glycemic and insulinemic responses following 30–60 min of exercise have been extensively studied, and a dose–response has been proposed between exercise duration, or volume, and improvements in glucose tolerance or insulin sensitivity. However, few studies have examined the effects of longer bouts of exercise in type 2 diabetes (T2D). Longer bouts may have a greater potential to affect glucagon, interleukin-6 (IL-6) and incretin hormones [i.e., glucagon-like peptide-1 (GLP-1) and glucose-dependent insulinotropic peptide (GIP)].

**Aim:**

To examine the effect of two bouts of long-duration, moderate-intensity exercise on incretins, glucagon, and IL-6 responses before and after exercise, as well as in response to an oral glucose tolerance test (OGTT) conducted the following day.

**Methods:**

Twelve men, six with and six without T2D, participated in two separate conditions (i.e., exercise vs. rest) according to a randomized crossover design. On day 1, participants either rested or performed two 90 min bouts of treadmill exercise (separated by 3.5 h) at 80% of their ventilatory threshold. All participants received standardized meals on day 1. On day 2 of each condition, glucose and hormonal responses were measured during a 4-h OGTT.

**Results:**

On day 1, exercise increased IL-6 at the end of the first bout of exercise (exercise by time interaction *p* = 0.03) and GIP overall (main effect of exercise *p* = 0.004). Glucose was reduced to a greater extent in T2D following exercise (exercise by T2D interaction *p* = 0.03). On day 2, GIP and active GLP-1 were increased in the fasting state (*p* = 0.05 and *p* = 0.03, respectively), while plasma insulin and glucagon concentrations were reduced during the OGTT (*p* = 0.01 and *p* = 0.02, respectively) in the exercise compared to the rest condition for both healthy controls and T2D. Postprandial glucose was elevated in T2D compared to healthy control (*p* < 0.05) but was not affected by exercise.

**Conclusion:**

Long-duration, moderate-intensity aerobic exercise can increase IL-6. On the day following exercise, fasting incretins remained increased but postprandial insulin and glucagon were decreased without affecting postprandial glucose. This long duration of exercise may not be appropriate for some people, and further research should investigate why next day glucose tolerance was unchanged.

## Introduction

Exercise recommendations for the prevention and treatment of type 2 diabetes (T2D) emphasize exercise prescriptions designed to target insulin sensitivity or body composition (1, 2). These outcomes have been extensively studied, and it is generally recognized that typical exercise-induced changes in body composition are modest and that changes in insulin sensitivity are short lived (1, 2). Evidence to support, adapt, and fine-tune these recommendations are rapidly accumulating. The most recent (November 2016) position statement of the American Diabetes Association on Physical Activity/Exercise and Diabetes (2) currently recommends:

- To enhance insulin action: daily exercise or at least not allowing more than 2 days to elapse between exercise sessions.
- For optimal glycemic and health outcomes: adults with T2D should ideally perform both aerobic and resistance exercise training.
- To prevent or delay the onset of T2D in populations at high risk and with prediabetes: structured lifestyle interventions that include at least 150 min/week of physical activity and dietary changes resulting in weight loss of 5–7%.

While exercise interventions based on this paradigm clearly contribute to meaningful reductions in the incidence of diabetes (3, 4) or hyperglycemia (5, 6), an unintended consequence of this success may have been a substantially smaller emphasis on the effects of exercise on other pathophysiologic disturbances present in T2D. For example, Defronzo (7) proposed an “ominous octet” of potential pathophysiologic targets that also includes an increased glucagon secretion and a decreased incretin effect. The effects of exercise on many of these other outcomes are largely unknown in people with T2D.

Insight regarding how exercise could potentially affect glucagon or incretins in T2D may be obtained from studies in other populations. For example, repeated long bouts (e.g., two bouts of 90 min) of moderate-intensity exercise performed on the same day have been shown to lead to reductions in glucagon and other counter-regulatory hormones, as well as reductions in sympathetic nerve activity, which persist until at least the next day in people with type 1 diabetes (T1D) (8) and in healthy participants (9). This has been studied as part of the concept known as hypoglycemia-associated autonomic failure or HAAF (10). Reduced glucagon responses may be problematic in T1D who can experience hypoglycemia in response to exercise or excess insulin and has been studied more extensively. However, T2D and impaired glucose tolerance are characterized by impaired postprandial suppression of glucagon and could potentially benefit from non-pharmacological reductions in glucagon (11–13).

Incretin hormones, such as glucagon-like peptide-1 (GLP-1) and glucose-dependent insulinotropic peptide (GIP), are secreted from the gastrointestinal tract into the portal circulation in response to nutrients. In a nutrient-dependent manner, incretins have been shown to contribute to lowering blood glucose by increasing insulin secretion, decreasing glucagon secretion, and decreasing the rate of gastric emptying [as reviewed by Drucker (14)]. On the other hand, GIP can increase glucagon secretion when glucose is low (15). The GLP-1 receptor has been found in cardiac muscle, smooth muscle of the vasculature, and perhaps skeletal muscle (16, 17). These findings, combined with the established heart rate (HR) increasing effect of GLP-1 (18), suggest that incretins could play a role in cardiometabolic responses to exercise.

Ellingsgaard et al. (19) have shown that increased interleukin-6 (IL-6) during exercise could stimulate secretion of GLP-1 from intestinal L cells and also pancreatic alpha cells. As reviewed by Pedersen (20), it is likely that increased circulating IL-6 during exercise is secreted directly by skeletal muscle and is proportional to the amount of glycogen depletion. *In vivo* human studies in healthy, obese, or T2D have often not observed an effect of exercise on incretin concentrations (21, 22), whereas a study in healthy runners observed increased GLP-1 following a marathon (23). It is unclear if this difference among human studies is due to differences in exercise duration or volume.

The effects of exercise clinical relevant outcomes such as glycated hemoglobin have been extensively studied (6, 24). The objective of this study was to examine the effect of two bouts of long-duration, moderate-intensity exercise on biomarkers, such as plasma glucagon, IL-6, GLP-1, and GIP. It was hypothesized that, compared to rest, two bouts of long-duration (i.e., 90 min) moderate-intensity exercise would increase plasma IL-6 and incretin hormone concentrations in T2D and in healthy participants. On the day after the exercise or rest conditions, participants returned to the laboratory for a 4-h oral glucose tolerance test (OGTT) and it was hypothesized that two bouts of long-duration moderate-intensity exercise would reduce the following day glucagon concentrations immediately before (fasted state) and during the OGTT. These objectives were examined using large amounts of exercise as a proof of concept with the understanding that this large amount of exercise (i.e., 3 h in a single day) is unlikely for most people and unsafe for some.

## Materials and Methods

### Research Design

**Figure 1:**
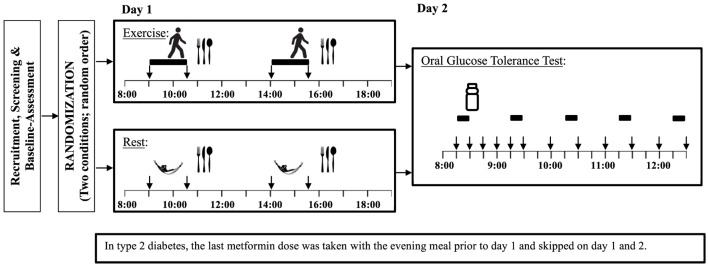
Study experimental design Legend: 
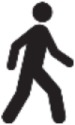
 = exercise, 
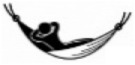
 = rest (Control), 
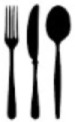
 = standardized meal, 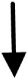 = blood samples, 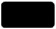 = heart rate variability and indirect calorimetry.

The experimental design involved two conditions that each required visits to the laboratory on two consecutive days (i.e., a total of four visits). On day 1 of each condition, participants were assigned to either exercise or control (i.e., rest) according to a randomized crossover design (Figure 1). On day 2 of each condition, participants return to the lab following an overnight fast for a 4-h OGTT. The 2-day exercise and control conditions were separated by at least 2 weeks.

### Participants

Twelve men, six without diabetes and six with physician diagnosed T2D, were recruited for this study. Men were selected since they have higher glucagon concentrations in response to various stimuli (e.g., exercise or hypoglycemia) (25) and previous exercise studies of this nature have shown larger reductions in counter-regulatory responses in men (22). To minimize heterogeneity in T2D and the risk of hypoglycemia with prolonged exercise, participants were required to be treated with lifestyle intervention(s) and metformin only. In order to be eligible, all participants also had to be non-smokers and not taking any beta-blockers. Furthermore, participants were excluded if they had cardiovascular or orthopedic limitations to exercise, or felt they would be unable to walk for 90 min without interruption. Many of the participants with T2D were recruited from our previous exercise studies (26, 27) and were purposefully identified due to their above average level of fitness as potential volunteers due to the long bouts of walking required in the present study. Comparable cohorts of men without diabetes had not previously been studied in our lab, therefore, recruited a convenience sample of healthy counterparts with similar body mass indices.

This study was carried out in accordance with the recommendations of the Tri-Council Policy Statement on “Ethical Conduct for Research Involving Humans” with written informed consent from all subjects. The protocol was approved by the University of Alberta Health Research Ethics Board.

### Baseline Assessment

Participants attended a baseline visit to measure glycated hemoglobin (A1c; DCA Vantage™ A1C Analyzer, Siemens Medical Solutions, Malvern, PA, USA), resting metabolic rate (RMR), and perform a graded submaximal exercise test with indirect calorimetry (TrueMax metabolic measurement system, Parvo Medics, Salt Lake City, UT, USA). HR was measured using a Polar heart rate monitor (Polar Electro, Finland). The submaximal exercise test was performed according to a modified Balke–Ware treadmill protocol where each participant walked at a self-selected speed, determined as comfortable but brisk, while the grade was increased by 1% each minute. The test was ended shortly after participants reached their individual ventilatory threshold (VT) using the V-slope criteria (28) as determined by a trained exercise physiologist.

Once eligibility was confirmed and baseline assessments were performed, a 1-month exercise habituation phase was completed by every participant that included three sessions of exercise per week at 80% of their VT. The duration began with 30 min and gradually progressed until participants could walk for 90 min continuously.

### Experimental Protocol

On day 1 of each condition, participants arrived at lab at 08h00 after a minimum 10-h overnight fast. They were asked to avoid vigorous exercise the day before each testing condition. An intravenous catheter was inserted into an antecubital vein and was kept patent with sterile saline. The exercise condition contained two 90-min bouts of treadmill exercise at intensity of 80% of the previously determined VT, as described in previous studies (9, 29). The first exercise bout began at 09h00 in the fasted state and the second at 14h00. Blood samples were taken immediately before and after each bout of exercise. Indirect calorimetry and HR measurements were collected during the first and last 10 min of each 90-min bout of exercise. During the non-exercise condition, participants remained sedentary but the above measures (except for HR) were collected at the same times as during the corresponding exercise condition.

The energy intake required to maintain energy balance during non-exercise condition was estimated based on participants’ previously measured RMR multiplied by a physical activity level (PAL) of 1.4 (Note: this PAL is typically used to characterize a sedentary lifestyle (30)). As in previous studies of this nature (31, 32), energy intake was kept the same on the exercise and non-exercise conditions. Energy intake was divided in two equal standardized meals (59% carbohydrate, 22% fat, 19% protein) provided 30 min after each exercise bout (see Figure 1). As such, the first exercise bout was performed in the fasting state and the second bout started 150 min after the first meal.

On day 2, participants returned to the lab after a minimum 10-h fast. Two fasting blood samples were taken; one 15 min before and the other immediately before the beginning of the OGTT containing 75 g of glucose (Trutol, Thermo Fisher Scientific, Canada). Ten blood samples were collected at specific time points following consumption of the glucose beverage (i.e., 15, 30, 45, 60, 90, 120, 150, 180, 210, and 240 min). Oxygen consumption and carbon dioxide production were collected for 10 min before the OGTT and for the last 10 min of each of the next 4-h periods (see Figure 1) using the same metabolic measurement system. Respiratory exchange ratio (RER) was determined as ratio between carbon dioxide production and oxygen consumption, while energy expenditure was calculated assuming non-protein energy equivalents. A metabolic equivalent (MET) was calculated as 1 kcal/kg/h (33). Participants were asked sit continuously throughout the test, with the exception of a bathroom break if required. Appetite rating and HR were collected during the same intervals. Appetite ratings were measured by a 150 mm visual analog scale and included questions on hunger, fullness, prospective food consumption, and desire to eat something sweet, salty, or fatty (34). HR was measured using a standard three-lead ECG over the same intervals as for the indirect calorimetry. Heart rate variability (HRV) is a tool that can be used to investigate the sympathetic and parasympathetic function of the autonomic nervous system. Autonomic nervous system activity can be affected by both hypo- and hyperglycemia. HRV indices included the root mean squared of the successive differences between R–R intervals (rMSSD), the SD of the R–R intervals (SDRR), and the ratio of low frequency spectral power to high frequency spectral power. For both day 1 and day 2, the first and last minutes of the 10-min indirect calorimetry and HR periods were excluded to allow for more stable data.

Participants with T2D refrained from taking their metformin dose on all four testing days. The last metformin dose was consumed more than 12 h prior to first blood sample which was taken on day 1 of each testing condition.

### Blood Samples

Each blood sample was first collected into a 10-mL EDTA vacutainer tube. Subsequently, 2.0 mL was transferred into a tube with 20 µL of a dipeptidyl peptidase (DPP-4) inhibitor (Millipore, MA, USA), 2.0 mL was transferred into a tube with 6.7 µL aprotinin (Millipore, MA, USA), and 0.25 mL whole blood was transferred into 1.0 mL ice-cold 8% perchloric acid. Aprotinin was added to inhibit proteases known to interfere with the determination of glucagon. The DPP-4 inhibitor was added to prevent degradation of active GLP-1. Perchloric acid was added to deproteinize the samples. The EDTA tubes were centrifuged at 1,500 × *g* for 10 min at 4°C. The tubes containing perchloric acid and aprotinin were centrifuged at 2,000 × *g* for 15 min at 4°C. Following centrifugation, the samples were immediately moved to a −80°C freezer until assays were completed.

Non-esterified fatty acids (NEFAs) were analyzed using commercially available kits (Wako Diagnostics, CA, USA), while plasma glucose and lactate were determined enzymatically using spectrophotometric assays. Total GIP, glucagon and insulin were measured using a Multi-Spot^®^ Assay System with a Sector^®^ Imager 2400 (Meso Scale Discovery^®^, MD, USA). Active GLP-1 was measured separately (Meso Scale Discovery^®^, MD, USA). Hematocrit was measured only on day 1 for both exercise and non-exercise conditions. Plasma IL-6 was also measured from day 1 plasma samples using a high-sensitivity ELISA (Quantikine HS human IL-6, R&D Systems Ltd., Abingdon, UK). Plasma metformin concentrations were assessed by high performance liquid chromatography in all plasma samples from day 1 as well as fasting samples from day 2. The concentration of phosphate solution used in the mobile phase was 20 mmol/L. The metformin assay was validated to a lower limit of quantitation of 8 ng/mL metformin based on 0.1 mL of human plasma (35). All assays were run in duplicate and the average of the two was reported.

### Statistical Analysis

The primary analyses were conducted using a three-way mixed factorial design ANOVA with *Diabetes* as a between group factor (i.e., T2D vs. healthy control), as well as *Exercise* (i.e., exercise vs. rest conditions) and *Time* (i.e., consecutive blood samples) as repeated measures factors. The number of levels for the time factor differed depending on the time period examined (e.g., day 1 had four consecutive blood samples). For day 2, The *Diabetes* by *Exercise* by *Time* ANOVA showed a significant effect of *Time* for all of the blood sample results (all *p* < 0.01). Therefore, it was deemed more informative to separate the fasting from the post glucose beverage results. For the 10 blood samples taken at different intervals postprandially, we considered both the area under the curve (AUC) and incremental AUC (iAUC). The AUC was calculated by the trapezoid method. The iAUC was calculated by subtracting the average of the two fasting values from the AUC. For the variables that were measured at 1-h intervals postprandially (e.g., calorimetry, HRV, and appetite) the four postprandial values were averaged. Age was a known confounder for HRV and was significantly associated with our HRV outcomes; we, therefore, considered age as a covariate for the statistical analyses on HRV. For each ANOVA, we examined interaction effects and main effects, but did not conduct the many possible *post hoc* comparisons due to lack of statistical power. Sphericity was tested using Mauchly’s test of sphericity. In the events where Mauchly’s sphericity test was significant the Greenhouse–Geisser correction was used.

Baseline characteristics were compared between groups using independent *t*-tests. Secondary analyses also examined the bivariate correlations among variables (e.g., IL-6 vs. GLP-1). Statistical tests were two-tailed, and *p*-values ≤ 0.05 were considered significant. Statistical analyses were performed with SPSS 21 (SPSS, Inc., Chicago, IL, USA).

## Results

### Participants

All 12 participants (6 T2D and 6 healthy) completed the study. Baseline characteristics are presented in Table 1. T2D and healthy participants had an average age of 60.5 ± 8.5 and 42.5 ± 10.5 years (*p* < 0.01) and an average body mass index (BMI) of 24.8 ± 4.3 and 26.7 ± 3.2 kg/m^2^ (*p* = 0.39), respectively. All T2D participants had a well-controlled glycemia as suggested by their A1c (6.4 ± 0.3%). They were treated with 500 to 1,500 mg of metformin per day and the average duration of diabetes diagnosis was 3.9 ± 2.3 years.

**Table 1:** Baseline characteristics.

	T2D	Healthy	*p*-Value
*n*	6	6	–
Age (years)	60.5 ± 8.5	42.5 ± 10.5	0.009
BMI (kg/m^2^)	24.8 ± 4.3	26.7 ± 3.2	0.39
Body weight (kg)	75.5 ± 16.2	81.6 ± 10.2	0.45
Duration of T2D (years)	3.9 ± 2.3	–	–
A1c (%)	6.4 ± 0.3	5.6 ± 0.1	<0.001
VO_2_@VT (mL/kg/min)	28.5 ± 5.6	37.2 ± 8.4	0.06
SBP (mmHg)	125 ± 14	131 ± 11	0.44
DBP (mmHg)	75 ± 10	71 ± 5	0.45

*T2D, type 2 diabetes; BMI, body mass index; A1c, glycated hemoglobin; NA, not applicable; NS, not significant; VT, ventilatory threshold; SBP, systolic blood pressure; DBP, diastolic blood pressure; RMR, resting metabolic rate*.

*Data presented as mean ± SD*.

### Day 1

#### Energy Expenditure and HR

All participants completed both 90-min exercise bouts without requiring adjustments to the exercise intensity. Indirect calorimetry and HR results from the exercise bout and corresponding rest conditions are presented in Table 2. As expected, energy expenditure and RER were significantly increased with exercise (main effect of *Exercise p* < 0.001). Energy expenditure corresponded to approximately one MET on the rest day and seven METs during exercise with no significant difference between T2D and healthy participants (see Table 2 for details). In addition to an increased RER with exercise, a significant *Exercise* by *Diabetes* interaction (*p* = 0.036) and an *Exercise* by *Time* interaction (*p* < 0.001) were observed for RER. These interactions were the result of RER being lower on the rest day in the healthy participants and after lunch in the exercise condition but greater after lunch in the rest condition. During the exercise bouts, HR averaged 121 ± 3 beats per minute during the first 10 min of each bout of exercise, or 72 ± 2% of age predicted maximum HR, and drifted upwards throughout exercise (main effect of time *p* = 0.001).

**Table 2:** Indirect calorimetry and HR at the beginning and at the end of two bouts of exercise or control (i.e., rest) on day 1.

		Healthy				T2D				*p*-Value
		9h00–9h10	10h20–10h30	2h00–2h10	3h20–3h30	9h00–9h10	10h20–10h30	2h00–2h10	3h20–3h30	
RER (VCO_2_/VO_2_)	Ex	0.87 ± 0.04	0.84 ± 0.02	0.90 ± 0.02	0.85 ± 0.03	0.89 ± 0.03	0.82 ± 0.03	0.89 ± 0.03	0.84 ± 0.04	Ex < 0.001
										Time < 0.001
	Rest	0.76 ± 0.03	0.74 ± 0.05	0.83 ± 0.03	0.80 ± 0.03	0.79 ± 0.04	0.79 ± 0.05	0.86 ± 0.05	0.84 ± 0.06	Ex × T2D = 0.036
										Ex × Time < 0.001
EE (METs)	Ex	7.25 ± 1.81	7.59 ± 1.65	7.29 ± 1.25	7.08 ± 1.14	6.78 ± 1.06	6.86 ± 1.01	6.56 ± 0.98	6.49 ± 1.19	Ex < 0.001
	Rest	0.93 ± 0.15	0.91 ± 0.11	1.12 ± 0.14	1.01 ± 0.10	0.86 ± 0.18	0.94 ± 0.11	1.08 ± 0.11	1.01 ± 0.16	
HR (bpm)	Ex	120 ± 13	135 ± 23	139 ± 23	145 ± 25	121 ± 6	139 ± 9	129 ± 9	141 ± 11	Time = 0.001
	Rest	NA	NA	NA	NA	NA	NA	NA	NA	

*Outcomes were measured during the first 10 min and last 10 min of each bout of exercise (or rest)*.

*T2D, type 2 diabetes; Ex, exercise; RER, respiratory exchange ratio; EE, energy expenditure; METs, metabolic equivalent (kcal/kg/h); HR, heart rate; bpm, beats per minute; NA, not available*.

*Data presented as mean ± SD. ANOVA examined main effect of exercise, diabetes, time, and their interactions. Only significant p-values are shown*.

#### Plasma Samples

**Figure 2:**
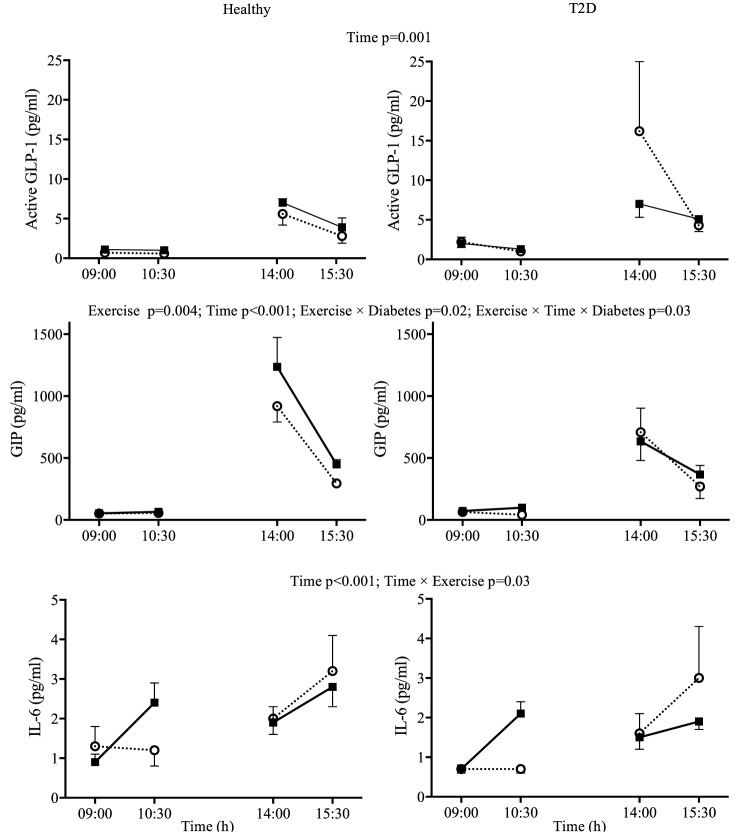
Day 1 plasma concentrations for interleukin-6 (IL-6), active glucagon-like peptide-1 (GLP-1), and glucose-dependent insulinotropic peptide (GIP) in response to two 90-min bouts of exercise (■) vs. rest (○) in healthy participants (left panels) and in type 2 diabetes (T2D) (right panels). Data shown as mean ± SEM.

Results of blood sample analyses from day 1 are summarized in Table 3 and Figure 2. There was a significant *Time* by *Diabetes* interaction (*p* = 0.03) suggesting that glucose changed to a greater extent in T2D over time. In addition, there was a significant main effect of *Exercise* leading to lower overall glucose concentrations in the exercise condition (*p* = 0.02).

**Table 3:** Concentrations of energy substrates and hormones before and after two 90-min moderate-intensity exercise bouts or rest on day 1.

		Healthy				T2D				*p*-value
		9h00 (pre-first bout)	10h30 (post-first bout)	2h00 (pre-second bout)	3h30 (post-second bout)	9h00 (pre-first bout)	10h30 (post-first bout)	2h00 (pre-second bout)	3h30 (post-second bout)	
Glucose (mmol/L)	Ex	4.7 ± 0.2	4.3 ± 0.3	4.8 ± 0.3	3.8 ± 0.2	6.1 ± 0.5	5.2 ± 0.2	8.8 ± 1.1	4.3 ± 0.3	Ex = 0.02
										Time, T2D < 0.001
	Rest	4.5 ± 0.3	4.7 ± 0.2	5.1 ± 0.8	4.5 ± 0.5	6.2 ± 0.6	6.2 ± 0.5	9.3 ± 1.1	6.9 ± 1.0	Time × T2D = 0.03
Lactate (mmol/L)	Ex	0.9 ± 0.2	1.1 ± 0.2	1.0 ± 0.2	1.0 ± 0.1	1.0 ± 0.1	1.2 ± 0.1	1.1 ± 0.1	1.2 ± 0.2	Ex × Time = 0.05
	Rest	1.0 ± 0.2	0.7 ± 0.0	1.1 ± 0.1	0.8 ± 0.1	1.0 ± 0.1	0.8 ± 0.1	1.2 ± 0.1	1.0 ± 0.1	
NEFA (mmol/L)	Ex	0.4 ± 0.1	1.2 ± 0.3	0.2 ± 0	1.1 ± 0.2	0.5 ± 0.1	1.4 ± 0.2	0.3 ± 0.1	1.1 ± 0.2	Ex, Time < 0.001
										Time × T2D = 0.02
	Rest	0.5 ± 0.1	0.5 ± 0.1	0.2 ± 0.0	0.3 ± 0.0	0.4 ± 0.1	0.6 ± 0.1	0.2 ± 0.0	0.2 ± 0.0	Ex × Time < 0.001
Insulin (pmol/L)	Ex	39.1 ± 8.9	20.8 ± 6.2	308.6 ± 81.7	23.9 ± 10.5	30.6 ± 9.5	36.3 ± 11.2	168.8 ± 48.5	37.7 ± 7.61	Time < 0.001
	Rest	37.2 ± 7.6	32.5 ± 8.9	319.9 ± 103	58.9 ± 21.2	31.3 ± 8.8	24.6 ± 8.3	392.9 ± 159.7	20.6 ± 42	
Glucagon (ng/L)	Ex	54.7 ± 6.5	81 ± 14.7	79.1 ± 5.5	114 ± 16.1	71.3 ± 6.7	78.9 ± 6.3	97.3 ± 19	103.6 ± 9.9	Ex, Time < 0.001
	Rest	50.2 ± 6.9	49.8 ± 7.7	77.7 ± 6.2	62.8 ± 6.6	58.9 ± 5.9	47.3 ± 9.3	86.4 ± 13.8	69.2 ± 4.6	Ex × Time = 0.02

*Outcomes were measured immediately before and immediately after of each bout of exercise (or rest). T2D, type 2 diabetes; Ex, exercise; NEFA, non-esterified fatty acids. Results presented as mean ± SEM. ANOVA examined main effect of exercise, diabetes, time, and their interactions. Only significant *p*-values are shown*.

There was a significant *Exercise* by *Time* interaction for IL-6 (*p* = 0.03). Visual Inspection of the graph in Figure 2 suggests that exercise increased IL-6 compared to rest when performed in the fasting state but not when performed after lunch when IL-6 was increased overall compared to fasting. The IL-6 responses were similar for participants with and without T2D (Figure 2). GIP followed a similar pattern during the first exercise bout compared to rest, but increased to a greater extent in healthy participants following lunch, leading to an *Exercise* by *Time* by *Diabetes* interaction (*p* = 0.03). Overall plasma metformin concentrations decreased from 402 ± 120 to 191 ± 52 ng/ml (main effect of *Time p* = 0.03) throughout day 1 and were not affected by exercise.

### Day 2

#### Energy Expenditure, HR, and Appetite

There were main effects of *Time* on energy expenditure and RER during the OGTT (both *p* < 0.001). There were no effects of *Exercise* or *T2D* on energy expenditure in the fasting state or postprandially. RER was greater in participants with diabetes but lower after exercise throughout day 2 (main effect of *T2D* and *Exercise*, both *p* ≤ 0.01 see Table 4). There was no statistically significant effect of exercise or diabetes and HRV indices during the OGTT. Overall, ratings for prospective food consumption were higher during the OGTT from the exercise condition compared to the rest condition (*p* = 0.03). However, postprandial fullness decreases following exercise in T2D only (Diabetes by Exercise interaction *p* = 0.04 for fasting; *p* = 0.056 for the mean postprandial values). Participants with T2D had a lower desire to eat something sweet in the fasting (*p* = 0.01) and postprandial state (*p* = 0.03), but a Diabetes by Exercise interaction (*p* = 0.045) indicated that exercise tended to increase the desire to eat something sweet in T2D while decreasing this rating in healthy participants during the OGTT.

**Table 4:** Indirect calorimetry and heart rate variability during fasting and following an oral glucose tolerance test on day 2.

		Fasting			Mean postprandial			ΔPostprandial		
		Healthy	T2D	*p*	Healthy	T2D	*p*	Healthy	T2D	*p*
RER (VCO_2_/VO_2_)	Ex	0.77 ± 0.04	0.73 ± 0.03	T2D < 0.01	0.82 ± 0.04	0.76 ± 0.02	T2D = 0.01	0.04 ± 0.03	0.03 ± 0.01	
	Rest	0.80 ± 0.03	0.76 ± 0.02		0.84 ± 0.03	0.80 ± 0.02	Ex < 0.01	0.05 ± 0.02	0.04 ± 0.02	
EE (METs)	Ex	0.85 ± 0.12	0.90 ± 0.11		0.91 ± 0.08	0.95 ± 0.12		0.05 ± 0.10	0.05 ± 0.05	
	Rest	0.85 ± 0.13	0.88 ± 0.08		0.91 ± 0.07	0.92 ± 0.11		0.06 ± 0.08	0.03 ± 0.07	
HR (bpm)	Ex	60 ± 5	59 ± 15		64 ± 4	61 ± 13		4 ± 2	2 ± 2	
	Rest	56 ± 5	61 ± 15		59 ± 4	62 ± 13		3 ± 1	1 ± 4	
RMSSD	Ex	51 ± 23	34 ± 15		40 ± 10	28 ± 11		−11 ± 12	−6 ± 10	
	Rest	60 ± 20	27 ± 14		49 ± 16	26 ± 11		−10 ± 5	−1 ± 9	
SDRR	Ex	80 ± 38	46 ± 17		66 ± 19	54 ± 26		−14 ± 23	8 ± 18	
	Rest	74 ± 25	46 ± 20		70 ± 20	48 ± 27		−1 ± 19	2 ± 13	
LF/HF	Ex	1.52 ± 1.14	0.91 ± 9.34		1.61 ± 0.72	1.32 ± 0.94		−0.04 ± 1.12	0.41 ± 1.17	
	Rest	0.77 ± 0.27	1.80 ± 1.03		1.27 ± 0.84	1.46 ± 1.28		0.53 ± 0.66	−0.34 ± 1.53	

*T2D, type 2 diabetes; Ex, exercise; RER, respiratory exchange ratio; EE, energy expenditure; METs, metabolic equivalent or kilocalories divided by kilograms of body mass and hours (kcal/kg/h); HR, heart rate; bpm, beats per minute; SDRR, SD of the R–R intervals; rMSSD, root mean squared of the successive differences between R–R intervals; LF/HF, the ratio of low frequency spectral power to high frequency spectral power; mean postprandial, average from 10 min at the end of each of the four 1-h postprandial periods; ΔPostprandial, mean postprandial minus fasting. Data presented as mean ± SD. ANOVA examined main effect of exercise, diabetes, time, and their interactions. Only significant *p*-values are shown*.

#### Plasma Samples

**Figure 3:**
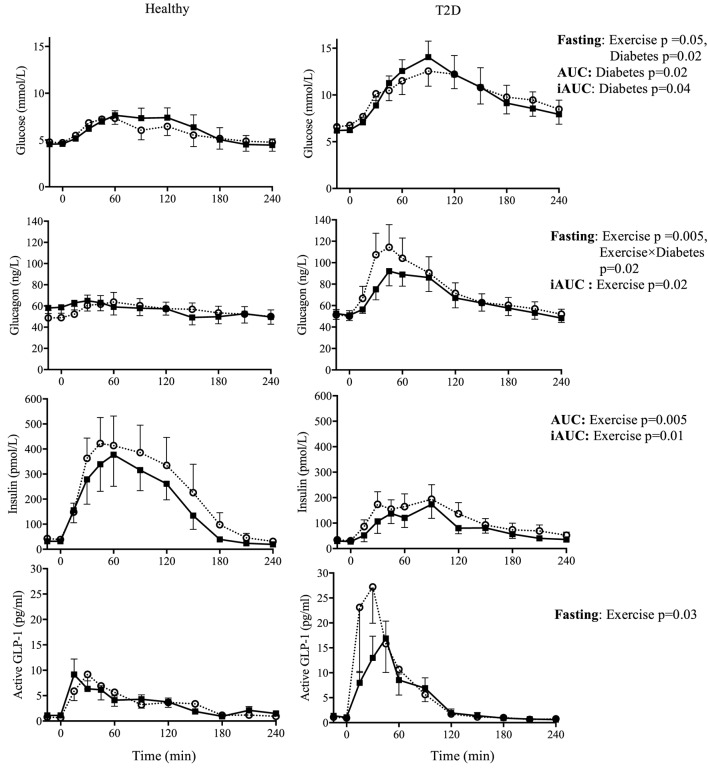
Day 2 fasting plasma concentrations (−15 and 0 min) and responses to an oral glucose tolerance test (area under the curve = AUC; incremental AUC = iAUC) for glucose, glucagon, insulin, and active glucagon-like peptide-1 (GLP-1), the day after two 90-min bouts of exercise (■) vs. rest (○) in healthy participants (left panels) and in type 2 diabetes (T2D) (right panels). Results from 2 × 2 ANOVA showing main effects of exercise vs. rest, diabetes vs. control, and their interaction. Data shown as mean ± SEM.

There were significant main effects of *Time* on all energy substrates and hormones on day 2. Therefore, analyses were conducted separately for the fasting and postprandial values. There was a main effect of *Diabetes* on fasting, AUC, and iAUC glucose (all *p* < 0.05) but no main effect of *Exercise* on the AUC and iAUC. However, there was a main effect of *Exercise* on fasting glucose (*p* = 0.05), with a 0.5 and 0.1 mmol/L decrease fasting glucose in the morning following exercise in the T2D and healthy control group, respectively (note: the *Diabetes* by *Exercise* interaction was not significant, *p* = 0.35), see Figure 3.

Exercise increased fasting glucagon in the healthy control group but not in T2D (*Exercise* by *Diabetes* interaction, *p* = 0.02), whereas the postprandial iAUC for glucagon was reduced by exercise (main effect of E*xercise, p* = 0.01). Fasting insulin was not affected by exercise but iAUC and AUC insulin were reduced (main effect of *Exercise, p* = 0.08, *p* = 0.01 and *p* = 0.001, respectively). In terms of the insulin:glucagon ratio, both the iAUC and AUC were reduced following exercise (main effect of *Exercise p* = 0.04 and *p* = 0.004, respectively), see Figure 3.

Fasting active GLP-1 and GIP concentrations showed a small increase with exercise (main effect of *Exercise*, both *p* < 0.05). Exercise on the previous day did not affect postprandial incretin hormones during the OGTT, see Figure 3 and Table 5.

**Table 5:** Concentrations of energy substrate and hormones during fasting and following an oral glucose tolerance test on day 2.

		Fasting			iAUC			AUC		
		Healthy	T2D	*p*	Healthy	T2D	*p*	Healthy	T2D	*p*
Glucose (mmol/L)	Ex	4.6 ± 0.1	6.2 ± 0.5	Ex = 0.05	372 ± 171	1,006 ± 264	T2D < 0.05	1,469 ± 190	2,499 ± 345	T2D < 0.05
	Rest	4.7 ± 0.2	6.7 ± 0.6	T2D < 0.05	269 ± 138	902 ± 193		1,408 ± 185	2,504 ± 304	
Lactate (mmol/L)	Ex	0.8 ± 0.1	0.8 ± 0	T2D < 0.05	38.8 ± 5.3	51.9 ± 11.5		221.4 ± 15.0	241.3 ± 9.8	Ex = 0.05
	Rest	0.7 ± 0.1	1.0 ± 0.1	Ex × T2D < 0.05	49.6 ± 7.4	26.6 ± 12.2		222.9 ± 10.0	264.8 ± 15.3	
NEFA (mEq/L)	Ex	0.7 ± 0.1	0.6 ± 0.1		−73.4 ± 23.2	−55.7 ± 8.9		90.3 ± 14.2	76.5 ± 13.1	
	Rest	0.6 ± 0.1	0.5 ± 0.1		−75.4 ± 12	−48.1 ± 6.5		66.7 ± 5.2	72.0 ± 17.0	
Insulin (pmol/L)	Ex	31.9 ± 7.6	28.7 ± 8.7		36,182 ± 9,974	13,992 ± 3,841	Ex = 0.01	43,836 ± 11,595	20,881 ± 5,203	Ex < 0.01
	Rest	40.7 ± 9.2	32.6 ± 8.7		47,004 ± 14,486	20,144 ± 5,078		56,777 ± 16,265	27,958 ± 6,422	
Glucose-dependent insulinotropic peptide (pg/mL)		59.9 ± 7.2	68.7 ± 10.7	Ex = 0.05	43,630 ± 6,230	34,329 ± 7,530		58,016 ± 5,804	50,827 ± 7,208	
		55.1 ± 6.4	57.2 ± 7.6		39,297 ± 6,526	36,856 ± 4,640		52,515 ± 5,716	50,594 ± 4,441	

*T2D, type 2 diabetes; Ex, exercise; AUC, area under the curve; iAUC, incremental area under the curve; T2D, type 2 diabetes; NEFA, non-esterified fatty acids. For all outcomes, there was a sigificant main effect of time. Data presented as mean ± SEM. ANOVA examined main effect of exercise, diabetes, and their interactions. Only significant p-values are shown*.

Upon arrival on day 2, fasting plasma metformin concentrations were very low and similar between exercise and rest (i.e., control) conditions (76 ± 17 to 83 ± 18 ng/ml, respectively, main effect of *Time, p* = 0.03).

#### Bivariate Correlations

There was no significant bivariate correlation between changes in IL-6, insulin or glucagon and changes in active GLP-1 or GIP, either when examining the participants with and without T2D together or separately on day 1. On day 2, there was an inverse association (*r* = −0.60, *p* = 0.038) between the exercise-induced changes in lactate and HRV as assessed by RMSSD. No associations were found between incretins and glucagon or insulin.

## Discussion

To our knowledge, no other study in T2D has examined the glycemic, hormonal, and metabolic responses to exercise of such a high volume in a single day (i.e., 3 h walking). Although other studies have suggested a dose–response relationship between exercise duration and improvements in glucose tolerance or insulin sensitivity (36–38), we did not observe any improvements in glucose tolerance following 3 h of exercise.

Unlike other studies in T2D or obesity which utilized shorter bouts of exercise (21, 22, 39), we observed elevated incretin hormones, particularly GIP, immediately after exercise (Figure 2). This increase persisted to the following day in the fasted state but not during the OGTT. It is unclear if these increases are practically meaningful as the increases were small in absolute terms and occurred at times when incretins were low. The increase was nonetheless consistent as we were able to detect these differences with a small sample size. In the participants with T2D who had relatively well-controlled glycemia, postprandial plasma incretin concentrations were not lower in T2D compared to healthy controls. While earlier studies suggested lower incretins in T2D, recent meta-analyses suggest that this is not always the case for both GIP (40) and GLP-1 (41). Another possibility to explain the strong incretin response (particularly for GLP-1) during the OGTT in our participants with T2D was that they were prescribed metformin, an oral hypoglycemic medication that has been shown to increase incretins (21). However, the last metformin dose had been consumed at least 36 h before the OGTT and metformin concentrations had been reduced less than 5% of the concentrations we observed in the hours following a morning dose of metformin (21). However, it is not known if the effect of long term metformin treatment could have persisted beyond 36 h.

According to Ellingsgaard et al. (19) an increased GLP-1 following exercise may be due to increased IL-6. Importantly, the increase in plasma IL-6 during exercise can be directly attributed to secretion from skeletal muscle and IL-6 is thought to be secreted in proportion to glycogen depletion [as reviewed by Pedersen (20)]. We observed that plasma IL-6 only increased compared to rest during the first exercise bout, which was performed in the fasting state and not during the second bout performed after lunch. The design of the present study does not allow us to conclude if the absence of an effect of exercise on plasma IL-6 after lunch was due to the meal itself or to a reduced effect when sequential exercise bouts are performed. However, a recent study found that consuming a carbohydrate beverage during 120 min of cycling abolished leg IL-6 release even though muscle glycogen was reduced to a similar extent compared to fasting exercise (42). IL-6 was also increased by lunch itself, which is consistent with previous studies (43, 44). Therefore, it appears that exercise-induced IL-6 secretion requires, or at least is more pronounced, with fasting exercise protocols.

A notable finding in the present study was that, in accordance with the hypothesis, two long bouts of exercise enhanced the postprandial suppression of glucagon (i.e., reduced iAUC). The postprandial suppression of glucagon is thought to be impaired in people with T2D (7). While both insulin and glucagon were lowered by exercise during the OGTT, insulin was reduced to a greater extent as reflected as a decrease in both the iAUC and AUC for the insulin:glucagon ratio. Insulin acts to suppress glucagon secretion; therefore, the observation of a lower glucagon in the presence of lower insulin is noteworthy since previous studies using hyperinsulinemic clamp protocols reported a reduced glucagon following exercise when insulin was maintained in the exercise and rest conditions (8, 9, 29). The mechanism by which this form of exercise suppresses postprandial glucagon concentration in T2D cannot be elucidated from this study and is indeed a topic of continued interest and debate (7, 45). Despite postprandial glucagon being reduced in our participants with T2D, postprandial hyperglycemia was not improved. While this may be disappointing from a clinical perspective, the similar concentrations of plasma glucose in both conditions may be considered fortuitous to examine changes in glucoregulatory hormones without needing to clamp glucose at a fixed concentration.

From a theoretical perspective, the absence of a glucose lowering effect of exercise during an OGTT performed on the following day in T2D was unexpected. It is generally believed that the glucose lowering effect of exercise is proportional to the duration or volume of exercise (37, 38). Although our study had a small sample size, the absence of the expected glucose lowering effect of exercise is unlikely to be due low statistical power as the post OGTT glucose AUC was slightly higher (1%) in the exercise condition. The reasons for the unchanged glucose were unclear, although postprandial insulin was reduced suggesting improved insulin sensitivity. Other studies have documented an absence of improvement in OGTT following longer bouts of exercise. For example, Tremblay et al. observed an increase in glucose AUC during an OGTT performed 16 h after 90 min of cycling at 67% of VO_2max_ (46). They attributed this increased to an increased adipose tissue lipolysis, increased NEFA, and a decreased glucose oxidation (46). This explanation is consistent with our observation of a decreased RER (i.e., indicating a decreased carbohydrate oxidation) and a tendency for increased NEFA (*p* = 0.09) during the OGTT from the exercise condition.

A primary limitation of the present study was the small sample size. As a result, our study was underpowered to detect potentially meaningful effects of exercise or diabetes. Our randomized crossover design helped to reduce the impact of this limitation on statistical power when comparing the exercise and rest conditions. However, the between participant comparison of T2D (*n* = 6) to healthy control (*n* = 6) was particularly underpowered for some outcomes.

The validity of conclusions regarding comparisons between T2D and healthy controls was further impaired by these small subgroups which were not matched for possible confounders (e.g., age, BMI, and fitness). BMI and exercise-induced energy expenditure ended up being relatively similar between the healthy and T2D participants; however, the healthy control group was younger and likely had different body composition (e.g., more fat free mass). Age was not associated with most outcomes in our study with the notable exception of HRV. HRV was lower in our T2D participants but these differences were no longer significance after adjusting for age. Although not statistically significant, there were trends to suggest an increase in indices of HRV following exercise in T2D but not in healthy participants. In addition to detecting differences in glucose, our study was able to detect other expected differences between healthy participants and T2D [e.g., RER (47) and glucagon (11)]. Nonetheless, the primary contributions to be retained from this article should be in regards to the exercise vs. control comparison.

The participants with T2D that were recruited for our study were likely more fit, more physically active, and leaner than many people with T2D. Such participants were selected to increase the likelihood of completing the exercise protocol and reduce the risk of injury. However, this selection also introduces potential bias. The phenotypic differences in our participants could influence many of the hormonal and metabolic responses to exercise. For example, participants with lower fitness or greater adiposity may have a different inflammatory profile or different inflammatory response to exercise (48, 49). This could potentially reduce generalizability of our result.

Another limitation of the study was the reliance on plasma concentrations of hormones taken from peripheral blood samples. These concentrations from the systemic circulation often do not reflect the exposure of other organs to these hormones (e.g., pancreas or liver). In addition, our multiplex hormone assay used has limitations in regards to specificity. For example, the glucagon assay has been shown to have some cross-reactivity with glicentin (or to a lesser extent oxyntomodulin) (50).

In conclusion, exercise can affect a variety of pathological features that can contribute to hyperglycemia. Potential benefits include decreasing postprandial hyperglucagonemia and increasing incretin concentrations. The exercise protocol (i.e., two 90 min bouts of exercise) used in this study is likely not feasible for most people. Larger samples sizes and closer matching of participant characteristics would be required to more carefully address differences between participants with normal glucose tolerance and those with T2D. Future studies should seek to better understand if similar results can be obtained with shorter exercise protocols, as well as the persistency of the observed changes.

## Ethics Statement

This study was carried out in accordance with the recommendations of the Tri-Council Policy Statement on “Ethical Conduct for Research Involving Humans” with written informed consent from all subjects. The protocol was approved by the University of Alberta Health Research Ethics Board.

## Author Contributions

NB, SRE, CS, MD, GP, PS, JL, and DB contributed to the conception of the study and obtained funding for this project. SRE, NB, and ÉM-C collected the data. SRE, NB, ÉM-C, KF, RG, and CD analyzed the data. SRE and NB drafted the manuscript, and all authors critically reviewed the manuscript to provide important intellectual content.

## Conflict of Interest Statement

The authors declare that the research was conducted in the absence of any commercial or financial relationships that could be construed as a potential conflict of interest.
